# Mapping Enzyme Activity on Tissue by Functional Mass Spectrometry Imaging

**DOI:** 10.1002/anie.201911390

**Published:** 2020-01-23

**Authors:** Brett R. Hamilton, David L. Marshall, Nicholas R. Casewell, Robert A. Harrison, Stephen J. Blanksby, Eivind A. B. Undheim

**Affiliations:** ^1^ Centre for Advanced Imaging, and Centre for Microscopy and Microanalysis The University of Queensland Brisbane QLD 4072 Australia; ^2^ Central Analytical Research Facility, Institute for Future Environments Queensland University of Technology Brisbane QLD 4001 Australia; ^3^ Centre for Snakebite Research & Interventions Liverpool School of Tropical Medicine Pembroke Place Liverpool L3 5QA UK; ^4^ Centre for Biodiversity Dynamics Department of Biology Norwegian University of Science and Technology 7491 Trondheim Norway; ^5^ Centre for Ecological and Evolutionary Synthesis Department of Bioscience The University of Oslo 0316 Oslo Norway; ^6^ Centre for Advanced Imaging, and Institute for Molecular Bioscience The University of Queensland Brisbane QLD 4072 Australia

**Keywords:** enzymes, functional assays, lipids, mass spectrometry imaging, PLA_2_

## Abstract

Enzymes are central components of most physiological processes, and are consequently implicated in various pathologies. High‐resolution maps of enzyme activity within tissues therefore represent powerful tools for elucidating enzymatic functions in health and disease. Here, we present a novel mass spectrometry imaging (MSI) method for assaying the spatial distribution of enzymatic activity directly from tissue. MSI analysis of tissue sections exposed to phospholipid substrates produced high‐resolution maps of phospholipase activity and specificity, which could subsequently be compared to histological images of the same section. Functional MSI thus represents a new and generalisable method for imaging biological activity in situ.

## Graphical abstract

**By bringing together** the specificity of enzymes for their substrates with mass spectrometry imaging, an approach has been developed that can reveal the location and efficacy of enzymatic activity in histological tissue sections with the analytical confidence of mass spectrometry detection. This approach is referred to as functional mass spectrometry imaging (fMSI).

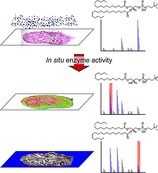

Enzymes are catalytic machines responsible for virtually every physiological process, and consequently, variations in enzyme abundance, activity, specificity, or efficacy are implicated in a wide range of pathologies.^1^, ^2^, ^3^, ^4^, ^5^ Although changes in enzyme abundance and activity can be quantified by assaying homogenised tissue, such methods are vulnerable to averaging effects, which obscure highly localised changes. High‐resolution spatially resolved assays of enzyme activity would therefore present a powerful tool for elucidating or diagnosing the causes of a range of diseases.^6^ Targeted imaging of enzyme activity using chemically labelled probes^6^, ^7^ or substrates has been demonstrated;^8^ however, no single imaging modality can routinely map the distribution of enzymes on tissue while simultaneously quantifying their catalytic efficacy and selectivity.

Mass spectrometry imaging (MSI)^9^ partially solves this problem through the sensitive detection and high‐resolution mapping of unlabelled analytes, such as spatial changes in metabolic profiles resulting from dysregulation of enzyme activity.^10^ However, conventional MSI approaches yield complex spectra that are limited by incomplete structural elucidation of detected compounds, and an inability to link a specific metabolite to a unique biochemical pathway. This ambiguity makes MSI‐based metabolomics an exhaustive approach for quantifying enzyme activity across tissues. Advanced MSI strategies that spatially resolve unique metabolites rely on the co‐deposition of a reactive substrate during tissue preparation.^11^, ^12^ This presents an opportunity to move beyond merely the localization of biomolecules, to mapping enzymatic activity. Here, we introduce functional mass spectrometry imaging (fMSI) for measuring the spatial distribution of enzymatic activity across tissues. We used the venom glands of the brown forest cobra (*Naja subfulva*) as our model system because its venom is known to be rich in in enzymatically active PLA_2_, making it ideal for the development of fMSI.^13^ We demonstrate that fMSI assays are well suited to profiling phospholipase A2 (PLA_2_) activity, and can therefore describe the spatial distribution of active PLA_2_ enzymes across histological sections. In principle, fMSI is transferrable to any MS‐based enzymatic assay, and is thus applicable to the visualisation of many biological processes across different tissue types with micrometer‐scale resolution.^14^

For the initial screening assay, separate solutions containing enzyme and substrate were mixed in vitro and infused directly into a mass spectrometer, which continually recorded the solution composition. To assay phospholipase activity, phosphatidylcholine (PC) substrates incorporating different fatty acids (FAs) on the glycerol backbone were selected. This enables discrimination between PLA_1_ and PLA_2_ activity, which facilitate selective ester hydrolysis at the *sn‐*1 and *sn‐*2 position, respectively.

As shown in Figure 1, the activity of porcine pancreatic PLA_2_ (ppPLA2) was measured on a pair of regioisomeric glycerophospholipids incorporating palmitic and oleic acid, denoted PC 16:0/18:1 and PC 18:1/16:0 (the X:Y nomenclature denotes the length and unsaturation of each FA^15^). A freshly prepared mixture of enzyme (in water) and substrate (in methanol) was infused directly into the mass spectrometer using a chip‐based nano‐electrospray ionization (nESI) source to eliminate sample carry‐over. Extracted ion chromatograms shown in Figure 1 A and 1 C reveal a temporal depletion of the intact substrate and a concomitant increase in products arising from catalytic lipid hydrolysis. The rate of substrate depletion by PLA_2_ is dependent on the length and degree of unsaturation of the FA.^16^ The major product observed upon mixing PC 16:0/18:1 with ppPLA_2_ (Figure 1 B) is lyso‐phosphatidylcholine 16:0 (LPC 16:0, green trace), arising from liberation of oleic acid from the *sn‐*2 position. The time‐dependent increase of a second product ion at *m*/*z* 522.4 (blue trace) was assigned to LPC 18:1. Based on MS/MS interrogation (Supporting Information, Figure S1), both species were assigned as LPCs arising from PLA_2_ activity on a mixture of isomers, rather than PLA_2_ and PLA_1_ activity on a unique substrate.^17^, ^18^ This hypothesis is consistent with the presence of PC 18:1/16:0 as an impurity in PC 16:0/18:1,^19^ as confirmed by regiospecific tandem mass spectrometry experiments (Supporting Information, Figure S2).^20^ A similar result was obtained for PC 18:1/16:0 in Figure 1 C and Figure 1 D, which demonstrates that PLA_2_ activity generates LPC 18:1 as the major product (blue trace), but also LPC 16:0 arising from the alternate regioisomer. Furthermore, the activity of ppPLA_2_ was trialled on various phospholipid substrates with similar results (e.g., PE 16:0/18:1, PC 16:0/22:6; Supporting Information, Figure S3), confirming the time‐dependent conversion of the PC substrate into LPC products. These data demonstrate the feasibility of MS approaches to simultaneously measure substrate depletion and product generation in real time and with high specificity.

**Figure 1:**
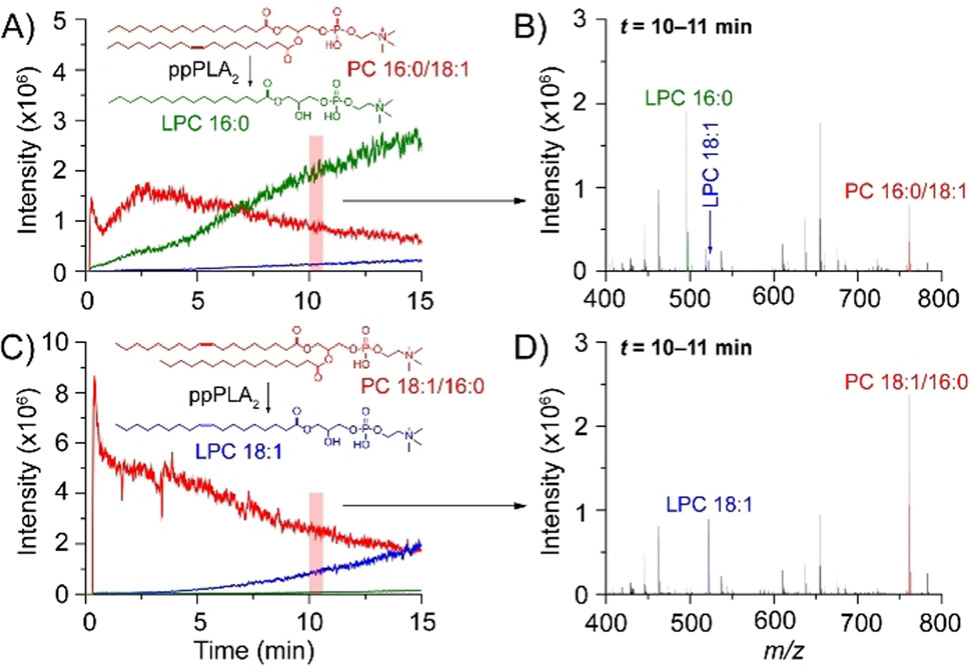
Mass‐spectrometry‐based enzyme assays are sensitive and powerful. Activity assay of porcine pancreatic PLA_2_ on phosphatidylcholine 34:1 isomers by nESI‐MS. A) The temporal change in abundance of the substrate (PC 16:0/18:1, red) and LPC products. B) Averaged mass spectrum from the shaded area in (A). C) Depletion of PC 18:1/16:0 (red), and formation of LPCs. Averaging over the shaded area yields the mass spectrum shown in (D).

The selectivity of PLA_2_ activity in milked *N. subfulva* venom was screened against PC substrates to unambiguously identify the LPC products of phospholipase activity (Supporting Information, Figure S4). This revealed that MALDI induced some formation of LPC from the PC substrates, although at a much lower rate than observed by PLA_2_ activity. After validating the assay in vitro, glycerophospholipid substrates were applied to tissue sections in order to spatially map phospholipase activity by matrix‐assisted laser desorption ionization (MALDI) mass spectrometry directly off the venom gland (i.e., fMSI). This experiment (Figure 2 D–F) revealed that product ions arising from phospholipase activity ([LPC 16:1+H]^+^, *m*/*z* 494.3, yellow) can be found across the venom gland, but with some notable regional variation, such as lower abundance and even small patches lacking PLA_2_ activity in some posterior parts of the gland. In contrast, the distribution of intact substrate ([PC 16:1/16:1+H]^+^, *m*/*z* 730.6, blue) is largely restricted to regions outside the tissue perimeter, where we also only observed extremely low LPC signal corresponding to a low amount of MALDI‐induced LPC formation. The identity of the major product ion in Figure 2 F was confirmed as enzyme‐generated LPC 16:1 by an analogous experiment using a matrix‐free tissue section and liquid extraction surface analysis coupled to a high‐resolution tandem mass spectrometer (Supporting Information, Figure S5).

**Figure 2:**
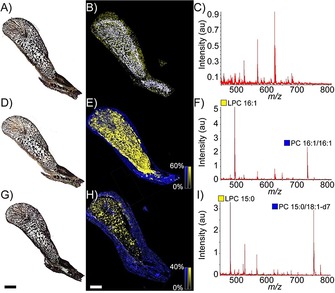
fMSI of *N. subfulva* venom gland, showing the distribution of PLA_2_ activity against two different substrates. A) Optical image of a 7 μm section of venom gland tissue. B) MALDI‐MSI ion map. C) Averaged MALDI mass spectrum in the absence of lipid substrate. Application of PC 16:1/16:1 (D–F) or PC 15:0/18:1‐d_7_ ((G)–(I)) with the MALDI matrix enables acquisition of fMSI ion maps of the substrate (blue) and PLA_2_ product (yellow) for each section ((E), (H)), along with their average spectra ((F), (I)). Scale bar: 2 mm.

To ensure that the product signals were not from endogenous LPCs, the fMSI experiment was repeated using a deuterium‐labelled substrate (PC 15:0/18:1‐*d*_7_; Figure 2 H). This experiment yielded significantly lower signal intensity than observed for PC 16:1/16:1, which is likely due to differences in the total amount of substrate deposited during sample preparation. Nevertheless, only the [*M*+H]^+^ ion corresponding to LPC 15:0 was observed (*m*/*z* 482.3; Figure 2 I), thus confirming that the products solely arise from PLA_2_ activity. Products associated with other phospholipases were not observed from *N. subfulva* venom (Supporting Information, Figure S4). Moreover, in the absence of applied substrate (Figure 2 B), no lipid signals were observed (Figure 2 C). Finally, adding a PLA_2_ inhibitor (Varespladib) prevented the formation of LPC 16:0 upon incubation of PC 16:0/18:1 with milked venom or liquid droplet extract from the venom gland of *N. subfulva* (Supporting Information, Figure S6). This was also the case in micro‐dissected samples from the venom gland, where we also confirmed the presence of enzymatically active PLA_2_ isoforms by bottom‐up proteomics (Supporting Information, Figure S6). This finding confirms the preservation of enzyme activity throughout histological sample preparation, and is consistent with an even distribution of PLA_2_ throughout the *N. subfulva* venom gland, as determined by shotgun proteomics of homogenized partitioned tissue sections (Supporting Information, Figure S7).

It is desirable to correlate the observed activity distribution with the spatial distributions of endogenous biomolecules, as well as histological features. The distribution of peptides and small proteins was therefore obtained by examining the same tissue section used for fMSI by conventional MSI (Figure 3). Venom PLA_2_ proteins were not detected in *N. subfulva* venom gland tissue by MSI, possibly because of their high molecular weight and low abundance relative to other venom components. Nonetheless, this analysis (Figure 3 C) revealed an intriguing non‐uniform distribution of masses corresponding to three‐finger toxins (3FTx) that has not previously been described. It is also worth noting that this heterogeneous distribution included a particular abundance of 3FTx in the same posterior region of the gland that showed little or no PLA_2_ activity (Figure 2). The functional significance of these findings remains to be elucidated; however, similar heterogeneous toxin distributions have been found in the venom glands of other animals, reflecting functionally distinct venoms^21^, ^22^ or evolutionary constraints.^23^ Following MSI acquisition and matrix removal, the tissue section can be stained using standard histological techniques (Figure 3 A). Thus, fMSI is compatible with conventional MSI and histological workflows in biomarker discovery.

**Figure 3:**
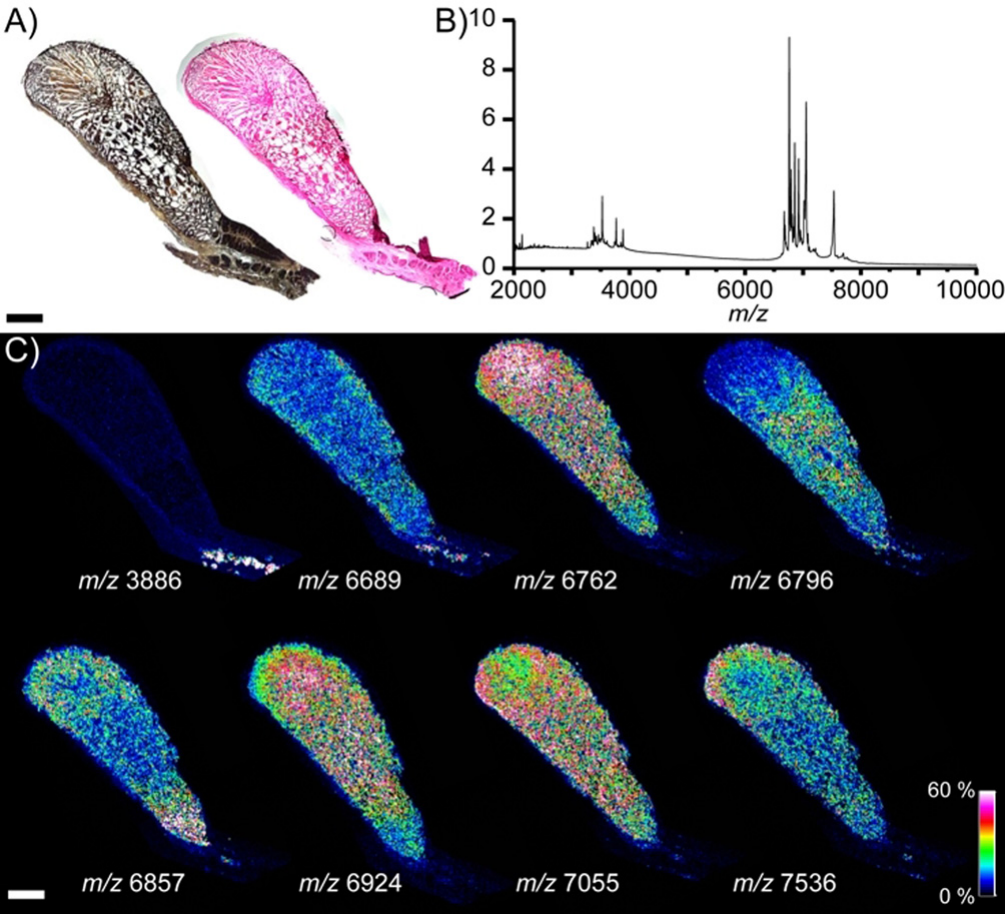
MSI and histology of the *N. subfulva* venom gland post‐fMSI. A) Section of the *N. subfulva* venom gland after paraffin removal before substrate and matrix deposition (left); an H&E stain obtained following fMSI and MSI data acquisition and matrix removal (right). B) Averaged mass spectrum from MSI analysis of the same *N. subfulva* venom gland section post‐fMSI acquisition, showing abundant signals from three‐finger toxins known to be abundant in the venom. C) Spatial distribution of endogenous compounds in the *N. subfulva* venom gland, including three‐finger toxins with possible cytotoxic and neurotoxic activities. Intensities of extracted ions (±1 Da) from normalized spectra are shown as heat maps across the tissue section. Scale bar: 2 mm.

These results greatly improve our knowledge of the basic biology of the snake venom gland because they demonstrate for the first time that PLA_2_ enzymes are functionally active during storage in the gland. The danger of venom enzymes to the glandular tissue is thought to be controlled by a variety of protein pre‐prodomain structures and the expression of enzyme inhibitors.^24^ This study suggests either that the glandular tissue is impervious to PLA_2_ degradation, or that the inhibitory system for controlling PLA_2_ bioactivity is readily disengaged, in this instance by histological processing. Either scenario raises fascinating questions regarding the co‐evolution of snake venom PLA_2_ and the factors that prevent the degradation of the venom gland in which they are stored.

There is a growing realisation of the versatility and power of MSI to map the distribution of specific biomolecules at micrometer‐scale resolution across tissue sections.^9^, ^10^, ^11^, ^12^, ^14^, ^25^ Functional MSI adds to the repertoire of these capabilities by providing, for the first time, a method to map the spatial distribution of enzymatic activity. By applying substrate onto tissue sections, the type and distribution of active enzymes can be mapped through their catalytic products. Although this study used PLA_2_ as the model enzyme, fMSI will be generally applicable to assaying the activity of different enzymes on any MSI platform, but particularly those equipped with high mass resolution and/or tandem mass spectrometry capabilities to increase confidence in product identification. Mass spectrometry is ideally suited to conducting simultaneous and comprehensive assays across multiple enzyme classes.^26^ The activities of many enzymes can also be reproduced using mimic substrates that are more suitable for analysis by mass spectrometry than native substrates because of their reduced molecular weight, greater solubility, and improved detectability.^27^ Taken together, fMSI will undoubtedly yield new insight into enzyme biochemistry, and the numerous roles of enzymatic activity in health and disease.

## Conflict of interest

The authors declare no conflict of interest.

## Supporting information

As a service to our authors and readers, this journal provides supporting information supplied by the authors. Such materials are peer reviewed and may be re‐organized for online delivery, but are not copy‐edited or typeset. Technical support issues arising from supporting information (other than missing files) should be addressed to the authors.

Supplementary
