# Supplementary material for: Mapping Enzyme Activity on Tissue by Functional Mass Spectrometry Imaging
Source: Angew Chem Int Ed Engl. 2020 Jan 23;59(10):3855–8. doi: 10.1002/anie.201911390 (PMC7106485; doi:10.1002/anie.201911390)
Supplement: Supplementary file 1 — Supplementary [file ANIE-59-3855-s001.pdf]

Angewandte Chemie
Eine Zeitschrift der Gesellschaft Deutscher Chemiker

Supporting Information

Mapping Enzyme Activity on Tissue by Functional Mass Spectrometry Imaging

Brett R. Hamilton, David L. Marshall, Nicholas R. Casewell, Robert A. Harrison, Stephen J. Blanksby, and Eivind A. B. Undheim**

anie_201911390_sm_miscellaneous_information.pdf

SUPPORTING INFORMATION — TABLE OF CONTENTS

METHODS **2**

Samples and standards 2

fMSI sample preparation 2

In vitro mass spectrometry-based PLA_2 assays 3

Isomer purity determination 4

Liquid Extraction Surface Analysis 4

MSI Acquisition 4

Histology 5

Bottom-up proteomics 5

FIGURES **7**

Figure S1. 7

Figure S2. 7

Figure S3. 8

Figure S4. 9

Figure S5. 10

Figure S6. 11

Figure S7. 13

REFERENCES **14**

SUPPORTING INFORMATION — METHODS

Samples and standards

Porcine pancreatic PLA_2 ($ppPLA_2$) was purchased from Sigma-Aldrich (Castle Hill, Australia; product number P6534-5MG). Phosphatidylcholine standards PC 16:0/18:1, PC 18:1/16:0, PC 16:0/22:6 and PC 15:0/18:1-d_7 were obtained from Avanti Polar Lipids (Alabaster, AL, USA). Solvents for mass spectrometry were Optima LC-MS grade (Thermo Fisher Scientific, Scoresby, Australia). CHCA was purchased from Sigma-Aldrich (Castle Hill, Australia; product number 70990-1G-F).

fMSI sample preparation

The brown forest cobra (*N. subfulva*; note that this species was until recently known as the forest cobra, *N. melanoleuca*) was a wild caught specimen of Cameroon origin housed in the herpetarium of the Liverpool School of Tropical Medicine – a UK Home Office approved and inspected experimental animal facility. Three days prior to euthanasian and venom gland dissection, venom was extracted and lyophilized prior to use. One venom gland from *N. subfulva* was fixed using RCL2 (Alphelys, France), and dehydrated through 70%, 90% and 100% ethanol (vol/vol), cleared with xylene, and embedded in paraffin. Tissue sections were cut and prepared as described previously.[1, 2] Briefly, sections were cut at 7 µm thickness from cold paraffin block using a microtome, placed directly onto an indium-tin oxide (ITO) glass slide, heated to 57 °C to attach the section, and the paraffin removed by repetitive xylene washes in order to improve sensitivity. This sample process avoids crosslinking of proteins, does not result in significant delocalisation of proteins[1, 2, 3] or loss of phospholipase activity (Fig 2; Supporting Information Fig. S5, S6). However, it removes endogenous lipids from the tissue sections, which is desirable when examining the enzymatic depletion of exogenous lipid substrates dosed onto the tissue.

For the application of PC substrates, stock solutions were first prepared by diluting 50 µL of 100 µM PC in 4 mL 50% methanol. A slide with two sections of the snake venom gland was placed into a vibrational vaporization-deposition system (Bruker ImagePrep), one covered with a glass coverslip and the other exposed. The PC stock solution was sprayed onto the tissue for 60 cycles, where each cycle included 2 s spray, 30 s incubation and 20 s drying time. At the completion of the PC application phase, the ImagePrep was thoroughly cleaned

with methanol and a CHCA solution (105 mg CHCA, 8 mL acetonitrile, 7 mL water, 30 µL trifluoroacetic acid) was applied using the standard Bruker ImagePrep CHCA application method to the control and PC-exposed snake venom gland sections.

In vitro mass spectrometry-based PLA_2 assays

An aqueous solution of $ppPLA_2$ (15 µL, 1 unit/mL) was added to 60 µL PC (3 µM in methanol, containing 5 mM ammonium acetate) in a 96-well PCR plate. 10 µL of the resulting solution was immediately sampled by a chip-based nano-electrospray ionization source (TriVersa NanoMate, Advion, Ithaca, NY, USA), which was used to eliminate carryover between isomeric samples, and infused into an LTQ Orbitrap Elite mass spectrometer (Thermo Fisher Scientific, Bremen, Germany) with a gas pressure of 0.5 psi (nitrogen) and a spray voltage of 1.4 kV. The mass spectrometer was operated in positive ion mode, at a mass resolution of 120,000 (at *m/z* 400), over a *m/z* range of 400 – 2000. Extracted ion chromatograms of substrates and plausible phospholipase products were assessed after stabilization of the electrospray (approximately 2 minutes).

MALDI-TOF analysis was undertaken using Flex Control 3.4 to operate an Autoflex Speed MALDI-TOF/TOF (Bruker, Bremen, Germany) in reflectron mode over a mass range of *m/z* 160 – 1400. 600 shots were acquired using a medium laser at 2000 Hz. CHCA was stored as a saturated solution in acetone, and diluted 10-fold into a solution of ethanol:acetone:0.1% trifluoroacetic acid (aq) (6:3:1 vol/vol) as a working matrix solution. 1 µL 100µM PC sample in methanol was mixed with 2 µL working matrix solution and spotted onto a polished steel target. Each PC substrate was analysed with and without milked *N. subfulva* venom. Acquired spectra were analysed using Flex Analysis 3.4 (build 76).

For confirming the enzymatic generation of LPC from PC substrates, PC16:0/18:1 was analysed as above with either milked venom, liquid droplet extraction (water), or laser micro-dissected (LMD) pieces from venom gland tissue sections, but with and without mixing venom or extract with 1 mM Varespladib (1:1 vol/vol). For micro-dissection of venom gland tissue, 10 µm sections were placed on PET membrane LMD slides (Leica), heated to 57 °C to attach the section, paraffin removed using xylene, and dissection carried out with an LMD-7 laser dissection microscope (Leica).

Isomer purity determination

The relative abundance of regioisomeric impurities in each synthetic PC standard was determined using a combination of collision- and ozone-induced dissociation to identify the fatty acyl moiety esterified at the *sn*-1 position of the glycerol backbone.[4] To facilitate this experiment, the LTQ Orbitrap Elite mass spectrometer is modified to enable the introduction of ozone into the ion trap *via* the helium bath gas, as previously described.[5, 6] Mass-selection and collisional activation (normalised collision energy = 65) of the $[PC+Na]^+$ ion generated by nESI yields an abundant $[PC+Na-183]^+$ ion. Re-isolation of this ion in the presence of ozone yields product ions carrying only the *sn*-1 fatty acid.

Liquid Extraction Surface Analysis

To determine if PLA_2 activity could be measured directly from tissue sections, deparaffinised tissue sections were loaded onto the sample stage of a robotically controlled micro-extraction and nESI system (Advion TriVersa NanoMate) operating in LESA mode. Using the robotic dispenser, a 1 µM solution of PC 16:1/16:1 in methanol was applied to a localised region of the tissue. After 5 s, the solvent was then re-aspirated and infused directly into a high-resolution MS (Thermo Fisher Scientific LTQ-Orbitrap Elite) *via* a chip-based nanoESI interface. Spectra were acquired at 120,000 resolving power (at *m/z* 400).

MSI Acquisition

fMSI data was acquired using Flex Imaging 4.1 and Flex Control 3.4 to operate a MALDI-TOF/TOF (Bruker Autoflex Speed) in reflectron mode over a mass range of *m/z* 160 – 1400. 600 shots were acquired using a medium laser spot size at 2000 Hz, resulting in a spatial resolution of 50 µm. For all samples, selected tissue-free regions were imaged as a negative control. After fMSI analysis, a linear-mode MALDI-TOF acquisition was undertaken over a mass range of *m/z* 1000 – 15276 at 2000 Hz, 50 µm spatial resolution, 600 shots, and using a medium laser size. Data was analysed post-acquisition using Flex Imaging 4.1 and SCILs LAB.

For acquisition of Varespladib-inhibited PLA_2 activity from tissue sections, we performed fMSI using an Atmospheric Pressure MALDI (AP-MALDI UHR, Masstech) coupled to a MicroTOF Q II QqTOf mass spectrometer (Bruker), which we found minimized MALDI-induced LPC formation. Tissue section of *N. subfulva* were prepared for fMSI as per above,

except that we applied 500 µM Varespladib in 50 % methanol (vol/vol) prior to PC substrate deposition. Varespladib solution was applied using the same protocol as during the PC substrate deposition by ImagePrep, with a total of 60 spray cycles, and partial deposition was achieved by covering parts of the section with a cover slip. Following the exposure to inhibitor, a solution of PC 16:0/18:1 (Avanti 850457C-200mg) was applied to the whole tissue using the same protocol as for the inhibitor for 60 cycles. The PC solution was 125 ug/mL in 50% methanol (aq). Following PC deposition, CHCA matrix was applied using ImagePrep as described above.

Acquisition on the AP-MALDI UHR MicroTOF Q II QqTOf was performed using Target ng 8.8.3 to control the AP-MALDI UHR and microTOF control 2.3 (build 40). The AP-MALDI was set to acquire in a vertical line movement, 80 µm pixel size, and 0.5 pixels per second. The microTOF Q II acquired 360 – 1000 Da at 0.5 seconds per scan, collecting only centroid data. Overall the data was acquired at 2 pixels per second. After the acquisition, the xml files with target ng raster information and the raw BAF file from the microTOF Q II were converted to an imzML file using MT imzML convertor (ng) version 1.0.1, which uses the MSconvert feature from Proteowizard 3.0.19228 (64 bit). The resultant imzML file was then imported into SCILS LAB MVS 2019c for visualisation. The normalisation applied was root mean squared (RMS). The PC substrate and LPC product masses were selected manually.

Histology

After fMSI or MSI experiments, CHCA matrix was removed using methanol, and then brought to an aqueous environment stepwise through 75%, 50%, and 25% methanol. The slides were then stained with Hematoxylin and Eosin using standard protocols described elsewhere.[1]

Bottom-up proteomics

To confirm the presence of PLA_2 on tissue and tissue micro-dissections, sections were treated with xylene to remove paraffin before they were either cut into equal parts or micro-dissected using a Leica LMD-7. Tissue was then dissolved in 50 mM ammonium bicarbonate 15 %

acetonitrile (vol/vol) pH 8, and cystines reduced by incubation in 5 mM dithiothreitol at 70 °C for 5 min before they were alkylated with 10 mM iodoacetamide at 37 °C for 90 min. The reduced and alkylated samples were then digested by incubating with 30 µg/µL trypsin overnight at 37 °C. The resulting tryptic peptides were desalted using a C18 ZipTip (ThermoFisher, USA), dried using vacuum centrifugation, dissolved in 0.5% formic acid (FA), and analysed by liquid chromatography tandem mass spectrometry (LC-MS/MS). Tryptic peptides from each quarter of venom gland section were analysed on a 5600 TripleTOF (Sciex, USA) equipped with a Turbo-V source heated to 550 °C. The dissolved samples were fractionated on a Shimadzu (Kyoto, Japan) Nexera UHPLC with an Agilent Zorbax stable-bond C18 column (Agilent, USA) (2.1 x 100 mm, 1.8 µm particle size, 300 Å pore size), using a flow rate of 180 µL/min and a gradient of 1–40 % solvent B (90 % acetonitrile [ACN], 0.1 % FA) in 0.1% FA over 60 minutes. MS1 spectra were acquired at 300–1800 m/z with an accumulation time of 250 ms, and selecting the 20 most intense ions for MS2. Precursor ions with a charge of +2 to +5 and an intensity of at least 120 counts/s were selected, with a unit mass precursor ion inclusion window of ± 0.7 Da, and isotopes within ± 2 Da were excluded. MS2 scans were acquired at 80–1400 m/z, with an accumulation time of 100 ms, and optimized for high resolution. Tryptic peptides from the LMD samples were analysed on an AB Sciex 6600 TripleTOF equipped with a Nanospray III source coupled to an Eksigent 400 nanoLC (Sciex, USA). 5 µL sample was loaded onto a trap column before it was separated with an Eksigent ChromXP-C18-CL column (300 µm x 15 cm, 3 µm particle size, 300 Å pore size), using a flow rate of 10 µL/min and a gradient of 1–40 % solvent B (90 % ACN, 0.1 % FA) in 0.1% FA over 60 minutes. MS1 spectra were acquired at 350–1800 m/z with an accumulation time of 250 ms, and selecting the 30 most intense ions for MS2. Precursor ions with a charge of +2 to +5 and an intensity of at least 200 counts/s were selected, with a unit mass precursor ion inclusion window of ± 0.7 Da, and isotopes within ± 2 Da were excluded for 6 seconds after MSMS event, which used rolling collision energy. MS2 scans were acquired at 100–1500 m/z, with an accumulation time of 100 ms, and optimized for high resolution.

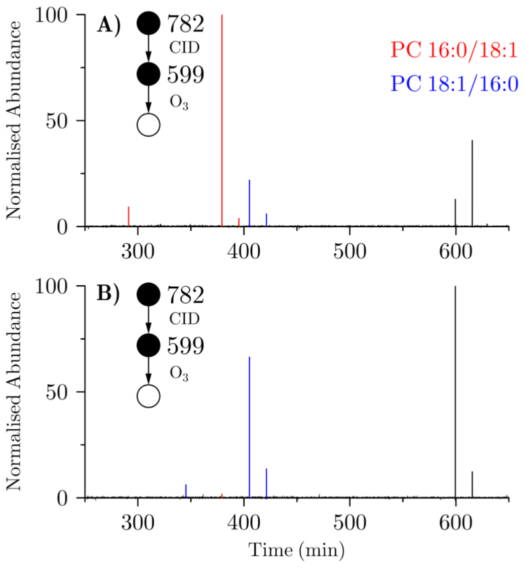

Figure S1. A combination of collision- and ozone-induced dissociation (OzID) of mass-selected $[M + Na]^+$ lipid ions reveals that both regioisomers of PC 16:0_18:1 are present in synthetic standards of **A**) PC 16:0/18:1 and **B**) PC 18:1/16:0.

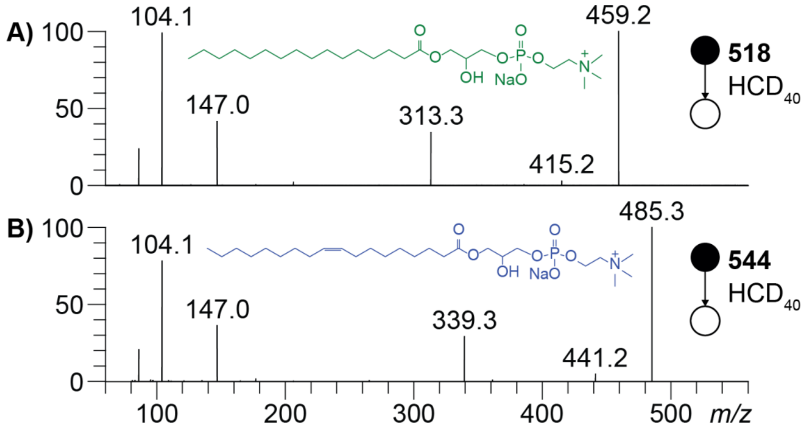

Figure S2. Tandem mass spectrometry (MS/MS) interrogation of mass-selected $[LPC + Na]^+$ ions by higher-energy collision-induced dissociation (HCD) confirms the LPC structure as a 1-acyl-2-hydroxyphosphatidylcholine (*i.e.*, hydrolysis of the ester at the *sn*-2 position). **A**) LPC 16:0 from addition of $ppPLA_2$ to PC 16:0/18:1; **B**) LPC 18:1 from $ppPLA_2$ treatment of PC 18:1/16:0. The ratio of product ions at *m/z* 104 and *m/z* 147 is diagnostic for the remaining esterification position.

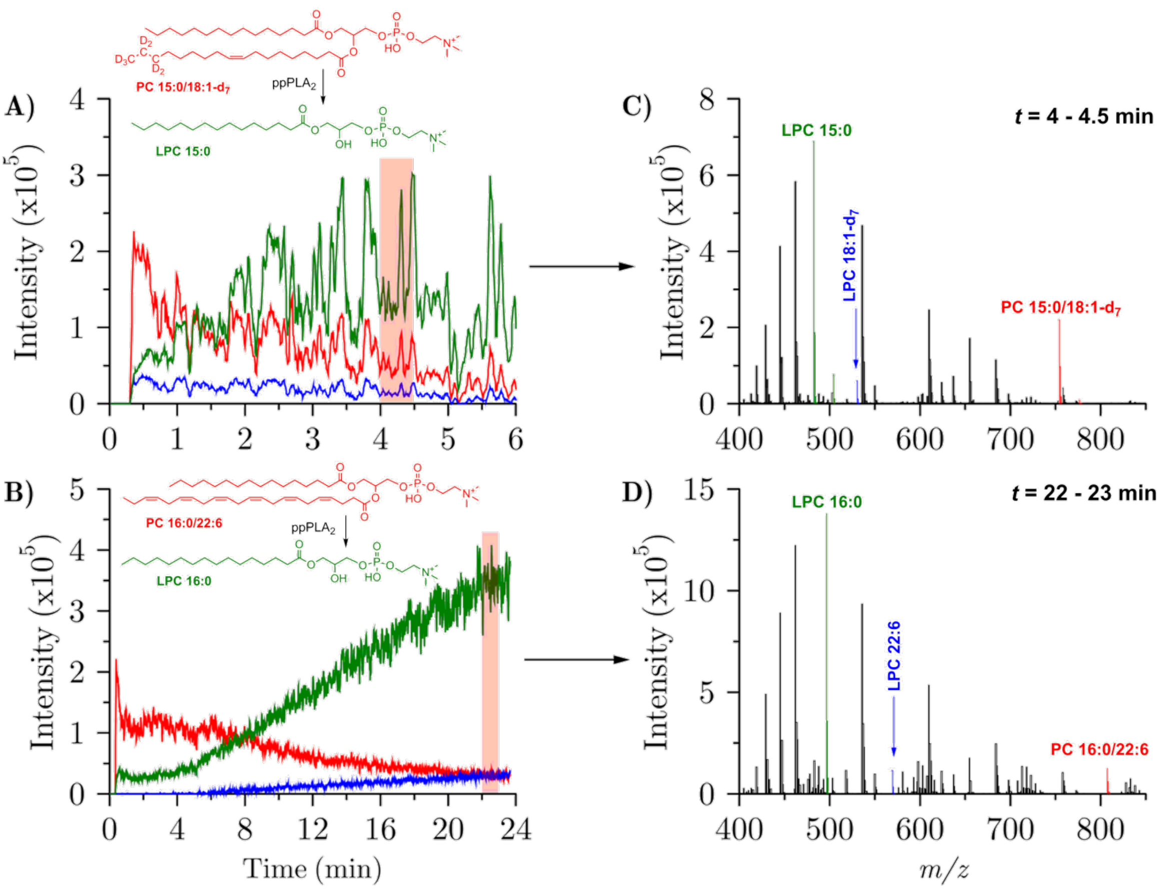

Figure S3. Activity assay of ppPLA$_2$ on PC substrates. Extracted ion chromatograms demonstrating PLA$_2$ activity on PC substrates. **A**) PC 15:0/18:1-d7 (*m/z* 753.6, red trace) and major products LPC 15:0 (*m/z* 482.3, blue trace) and LPC 18:1-d7 (*m/z* 529.4, green trace). **B**) PC 16:0/22:6 (*m/z* 806.6, red trace) and major products LPC 16:0 (*m/z* 496.3, blue trace) and LPC 22:6 (*m/z* 568.3, green trace). **C**, **D**) show the averaged mass spectra from time points indicated by the shaded areas in **A** and **B**, respectively. Data obtained using a high-resolution mass spectrometer (LTQ Orbitrap Elite, Thermo Scientific) with a nESI interface (TriVersa NanoMate, Advion).

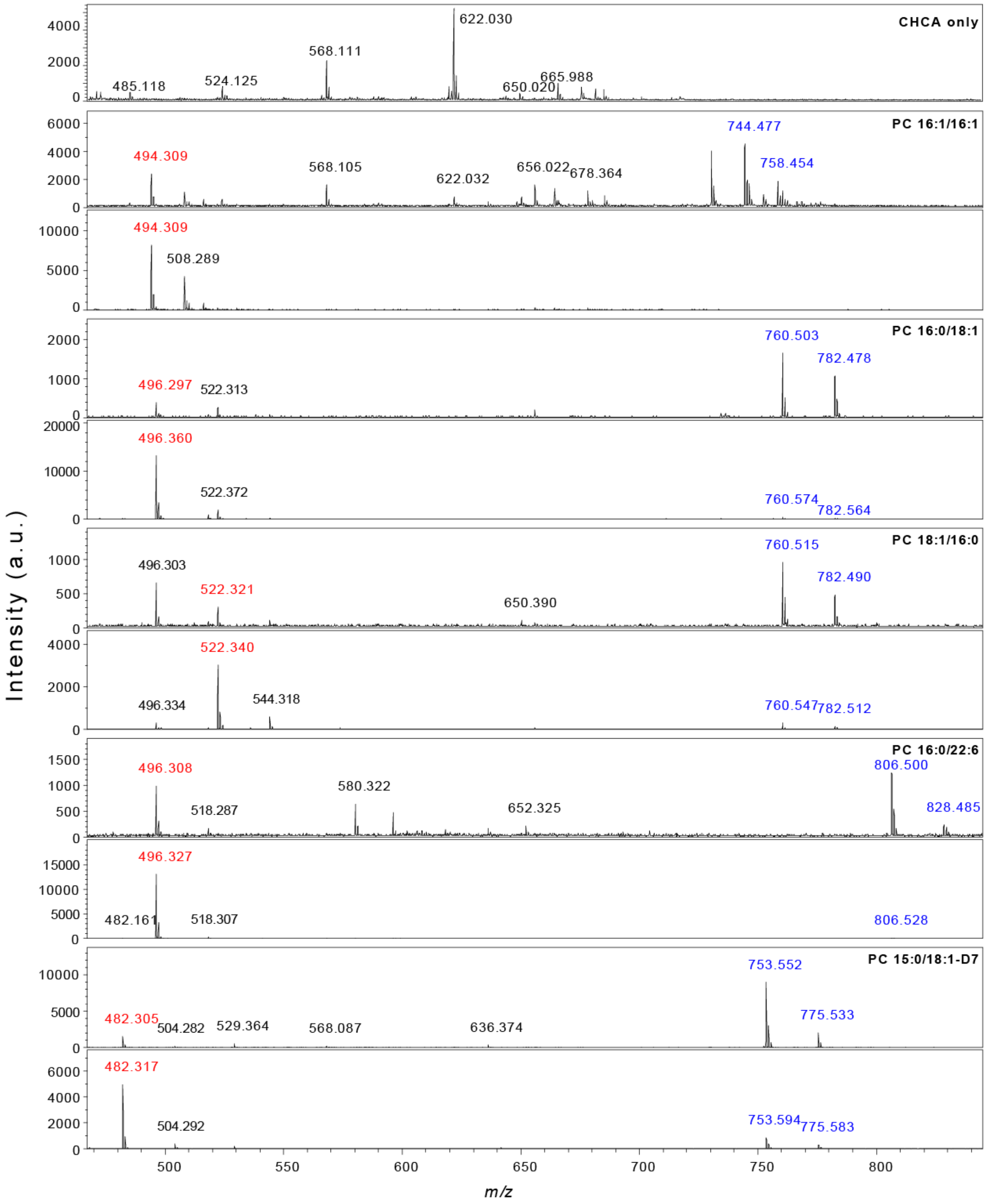

Figure S4. The milked venom of *N. subfulva* contains active PLA_2. MALDI-MS spectra acquired in positive reflectron mode (Autoflex Speed MALDI-TOF/TOF mass spectrometer) of a range of PC substrates spotted without (upper) and with (lower) venom from *N. subfulva*. PC substrate is specified in top right corner of each pair, *m/z* values corresponding to substrate is shown in blue, while red text denotes LPC products resulting from PLA_2 activity. Note that minor LPC products are observed in the absence of venom due to acidic sample preparation protocols. Top spectrum is matrix-only (CHCA).

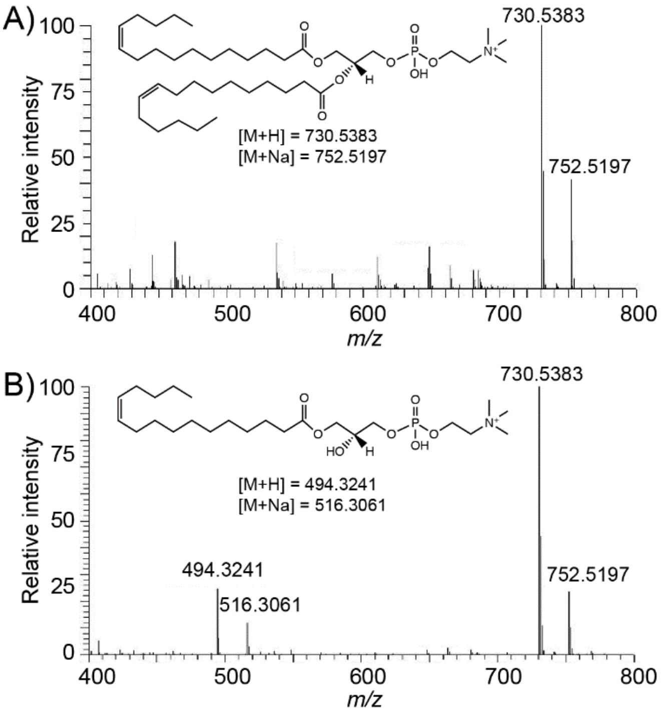

Figure S5. Enzyme (PLA_2) activity can be measured on tissue sections by LESA-MS (Advion TriVersa NanoMate coupled with a Thermo Fisher Scientific LTQ Orbitrap Elite mass spectrometer) to validate on-tissue MS-based enzyme assays. Using robotic sampling handling and a single-use pipette tip, PC 16:1/16:1 in methanol/chloroform (1:1, vol/vol) is deposited onto a venom gland section from *N. subfulva* mounted on a glass slide. After 10 seconds exposure, the solvent is re-aspirated and held in the tip for 20 seconds before infusion into the mass spectrometer via the nESI chip. LESA-MS spectra from **A**) a region of the slide with no tissue present; **B**) a region of the venom gland section. Micro-extraction sample area *ca*. 1 mm^2.

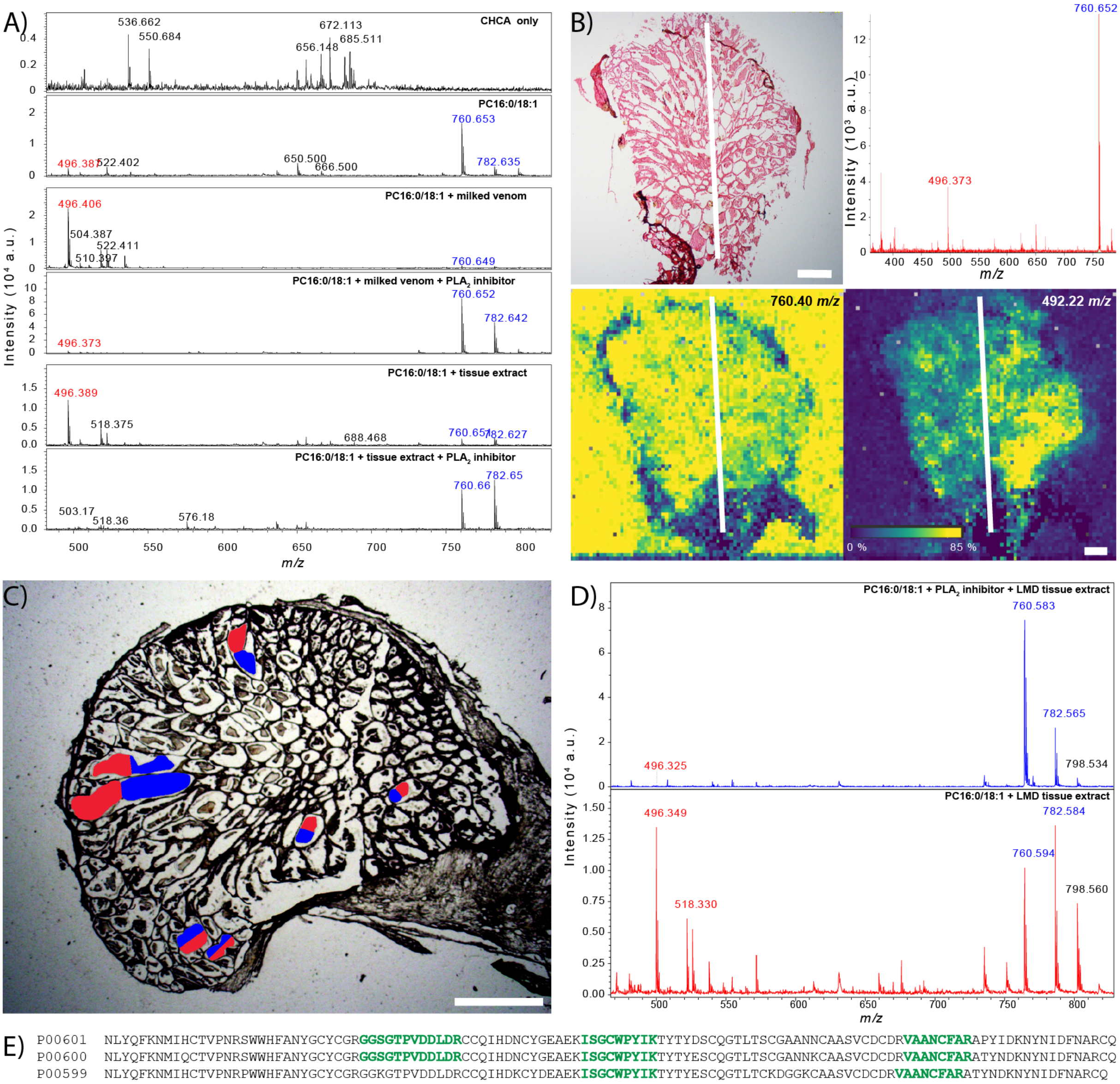

Figure S6. The formation of LPC is primarily due to activity of *N. subfulva* venom PLA$_2$. **A**) MALDI-MS spectra acquired in positive reflectron mode (Autoflex Speed MALDI-TOF/TOF mass spectrometer) of PC 16:0/18:1 substrate spotted with milked venom (middle pair) or venom gland extract (lower pair), without (upper) or with (lower) the PLA$_2$ inhibitor Varespladib. *m/z* values corresponding to substrate is shown in blue, while red text denotes the LPC product resulting from PLA$_2$ activity. Note that minor LPC products are observed in the absence of venom due to acidic sample preparation protocols. **B**) Spraying Varespladib on part of the venom gland (left side of white line) prior to the deposition of PC substrate resulted in suppression of PLA$_2$ activity as determined using an AP-MALDI UHR source coupled to a MicroTOF Q II QqTOf mass spectrometer. The same section stained with hematoxylin and eosin is shown top left, the normalized average spectrum with the peaks

corresponding to PC substrate (blue) and LPC product 16:0 (red) is shown the top right, while their distributions are shown as heat maps below. **C**) To further confirm that the LPC formation was primarily due to PLA_2 enzyme activity, we used a Leica LMD-7 laser dissection microscope to collect approximately equal halves of contents within the ductules of a subsequent venom gland section (red or blue shapes in micrograph on left), which ensured equal content between the two groups. Each of the two groups of micro-sections were pooled and then incubated with PC 18:1/16:0, with (top) or without (bottom) 500 µM Varespladib. This revealed complete inhibition of LPC product formation by Varespladib, suggesting that incomplete suppression of LPC product formation observed in **B**) was likely due to problems with inhibitor application rather than an inability by Varespladib to prevent LPC formation. *m/z* of peaks corresponding to PC substrate and LPC product are indicated in blue and red, respectively, while the colours of the spectra correspond to the colour-coded micro-dissections in **C**). **E**) To confirm the presence of PLA_2 in the micro-dissection sample inhibited by Varespladib, we analysed the remaining solution by bottom-up proteomics, which indeed identified known enzymatically active venom PLA_2 from *Naja melanoleuca*, which is a closely related sister species to *N. subfulva*. UniprotKB accession numbers are given for each PLA_2, while amino acid sequence regions corresponding to high-confidence matches in our analysed tryptic digest are shown in bold green. P00599 is Basic phospholipase A_2, P00600 is Acidic phospholipase A_2 DE-II, and P00601 is Acidic phospholipase A_2 DE-III.

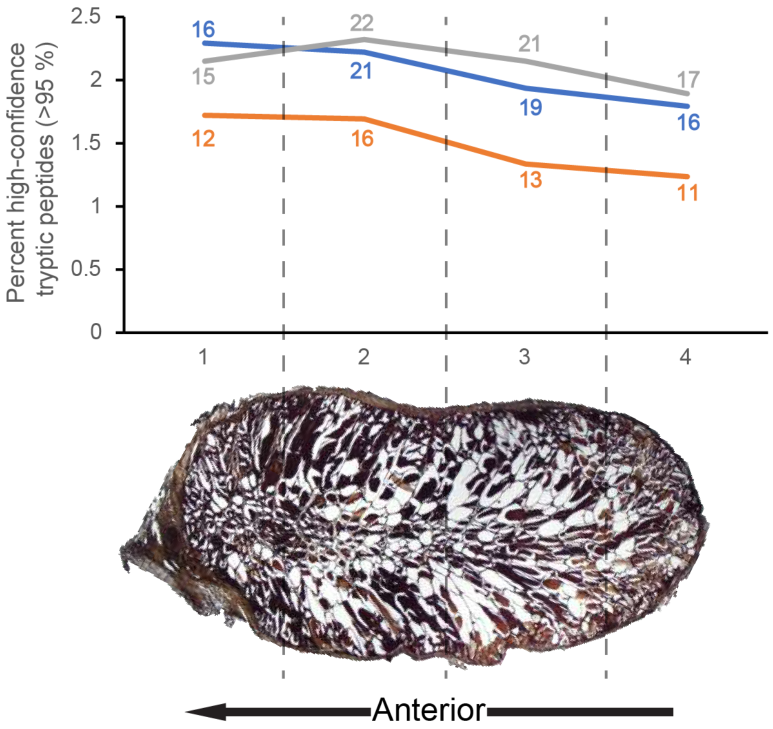

Figure S7. Venom PLA_2 is found along the whole length of the venom gland of *N. subfulva*. A section of *N. subfulva* venom gland (below) was dissected into four parts, soluble protein dissolved in water, and analysed by shotgun LC-MS/MS. The graph (top) shows the number of unique trypsin-digested peptide fragments confidently assigned by Protein Pilot (>95 %) to three PLA_2 characterised from the venom of the closely related *N. melanoleuca*, normalized to the total number of high-confidence peptides assigned to all proteins from *Naja* spp. The absolute number of high-confidence peptides is shown in colours corresponding to each PLA_2. Orange indicates basic PLA_2 (UniProt accession P00599), grey and blue indicate acidic PLA_2's (UniProt accessions P00600 and P00601, respectively).

SUPPORTING INFORMATION — REFERENCES

[1] E. A. B. Undheim, B. R. Hamilton, N. D. Kurniawan, G. Bowlay, B. W. Cribb, D. J. Merritt, B. G. Fry, G. F. King, D. J. Venter, *Proc. Natl. Acad. Sci. U.S.A.* **2015**, 112, 4026–4031.

[2] E. A. B. Undheim, K. Sunagar, B. R. Hamilton, A. Jones, D. J. Venter, B. G. Fry, G. F. King, *J. Proteomics* **2014**, 102, 1–10.

[3] J. M. Surm, H. L. Smith, B. Madio, E. A. B. Undheim, G. F. King, B. R. Hamilton, C. A. van der Burg, A. Pavasovic, P. J. Prentis, *Mol. Ecol.* **2019**, 28, 2272–2289.

[4] H. T. Pham, A. T. Maccarone, M. C. Thomas, J. L. Campbell, T. W. Mitchell, S. J. Blanksby, *Analyst* **2014**, 139, 204–214.

[5] D. L. Marshall, H. T. Pham, M. Bhujel, J. S. R. Chin, J. Y. Yew, K. Mori, T. W. Mitchell, S. J. Blanksby, *Anal. Chem.* **2016**, 88, 2685–2692.

[6] M. R. L. Paine, B. L. J. Poad, G. B. Eijkel, D. L. Marshall, S. J. Blanksby, R. M. A. Heeren, S. R. Ellis, *Ang. Chem.* **2018**, 130, 10690–10694.
